# Outcomes of implementation of the FilmArray meningoencephalitis panel in a tertiary hospital between 2017 and 2020

**DOI:** 10.1371/journal.pone.0265187

**Published:** 2022-03-17

**Authors:** TeeKeat Teoh, James Powell, Jillian O’Keeffe, Eoghan Donlon, Lisa Dillon, Marie Lenihan, Amanda Mostyn, Lorraine Power, Peter Boers, Patrick J. Stapleton, Nuala H. O’Connell, Colum P. Dunne

**Affiliations:** 1 Department of Clinical Microbiology, University Limerick Hospital Group, Limerick, Ireland; 2 Centre for Interventions in Infection, Inflammation & Immunity (4i), Limerick, Ireland; 3 School of Medicine, University of Limerick, Limerick, Ireland; 4 Department of Neurology, University Limerick Hospital Group, Limerick, Ireland; Aga Khan University - Kenya, KENYA

## Abstract

**Background:**

Acute meningoencephalitis is encountered commonly in the acute hospital setting and is associated with significant morbidity and mortality, in addition to significant healthcare costs. Multiplex PCR panels now allow syndromic testing for central nervous system infection. The BioFire® FilmArray® Meningoencephalitis (ME) allows testing of 14 target pathogens using only 0.2mls of cerebrospinal fluid (CSF). We conducted a retrospective observational study to assess the performance of the assay and secondarily to observe the clinical utility of negative results by comparing clinical outcomes of aseptic meningitis to bacterial and viral meningoencephalitis.

**Methods:**

Data for CSF samples tested using the FilmArray ME panel from October 2017 to October 2020 were analysed. Detection of bacterial and viral targets was analysed. Admission to critical care area, 90-day readmission rates, average length of stay and 30-day and 90-day mortality were analysed for three groups with following diagnoses: bacterial meningitis, viral meningoencephalitis, or aseptic meningitis.

**Results:**

From October 2017 to October 2020, 1926 CSF samples were received in the Clinical Microbiology laboratory. Of those, 543 CSF samples from 512 individual patients were tested using the FilmArray ME panel. Twenty-one bacterial targets and 56 viral targets were detected during the study period. For viral targets, the cumulative specificity was 98.9% (95% confidence interval: 93.1–99.9) when compared to the reference laboratory methods. The outcomes for 30- and 90-day mortality of the aseptic meningitis group were non-inferior relative to the viral meningoencephalitis and bacterial meningitis group. Patients with bacterial meningitis had a longer average length of stay. Aseptic meningitis was associated with a higher 90-day readmission rate than the other 2 groups, but without statistical significance.

**Conclusion:**

In our hands, implementation of the FilmArray ME panel was relatively straightforward. We experienced a transition in our workflow processes that enabled streamlining of CSF diagnostics and the safe removal of Gram staining in those samples being tested by this molecular assay. Coupled to this improvement, there was a positive clinical impact on patient care due to rapid turnaround time to results.

## Background

Acute meningoencephalitis is encountered commonly in the acute hospital setting. In the United States, 26,429 cases of meningitis or encephalitis were identified between 2011–2014, with an overall mortality of 2.9% [1]. Bacterial meningitis remains associated with relatively high mortality and, often, long-term neurological sequelae [2–4]. Globally, bacterial meningitis has been estimated to result in the loss of 21.87 million disability adjusted life years between 1990 and 2016 [3]. In Ireland, despite a reduction in invasive meningococcal disease in the last 2 decades, there is still 1.9 notified cases per 100,000 population and 1.3 cases per 100,000 population for all other bacterial meningitis [5–7]. Ireland remains to have the highest notification rate of invasive meningococcal disease in Europe [8]. Furthermore, even though culture and Gram stain are used routinely in laboratories, there is no consensus gold standard for laboratory diagnosis of bacterial meningitis and only estimated incidences are available. Nonetheless, in bacterial meningitis, a high level of clinical suspicion, combined with appropriate early diagnostics and empiric antibacterial agents can reduce unfavourable outcomes in patients who present with symptoms suggestive of bacterial meningitis.

Although outcomes for uncomplicated viral meningitis are excellent, and the illness is self-limiting in a majority of cases, viral meningitis nonetheless leads to hospitalisation for many patients. A 2018 report estimated annual United Kingdom incidence of viral meningitis in adults as 2.73 cases per 100,000 population [9]. Viral encephalitis is rarer, but results in more significant long-term sequalae and mortality, especially for certain viral pathogens such as herpes simplex virus (HSV) and Japanese encephalitis virus. Acute encephalitis has been estimated to cost the UK’s National Health Service over £23 million per year, but incidence is likely to be underestimated [10].

In addition to infectious causes of meningoencephalitis, immune-mediated or other aetiologies also add to the complexity of the diagnosis for patients presenting with symptoms consistent with acute meningitis or encephalitis. In a Canadian study, Parpia et al. reported that 51% of all known encephalitis cases had an unidentified aetiology while 27.7% had viral causes [11]. Aseptic meningitis, defined as patients having clinical and laboratory evidence for meningeal inflammation, has been reported to occur in 7.6 per 100,000 adults [12]. Shukla et al. reported that 81% of patients with aseptic meningitis had indeterminate aetiologies despite further investigations [13]. Conversely, McGill et al. concluded for their cohort study that unnecessary antiviral treatment was associated with longer hospital stays and that rapid diagnostics leading to rationalisation of treatment may reduce the burden of meningitis on health services [5]. Diagnostic accuracy in the examination of cerebrospinal fluid, of which often only small volumes are obtained, is essential due to the potential severity of acute meningoencephalitis. In that context, it is usual that only single target polymerase chain reaction (PCR) for specific pathogens, particularly HSV, would be available albeit complemented by microscopy and bacterial culture. However, the advent of nested or multiplex PCR panels heralded emergence of syndromic testing for central nervous system (CNS) infections.

The BioFire® FilmArray® Meningoencephalitis (ME) (bioMérieux, Marcy l’Étoile, France) panel has been of particular interest to many clinical microbiology laboratories due to its ease of use, ability to detect 14 pathogens using only 0.2mls of CSF, previously published sensitivity of 90%, 97% specificity, and negative predictive value of 98.7% [14]. While there is a requirement for capital investment, Soucek et al. suggested that even for a relatively small community hospital, such funding could be offset effectively through significant savings in antibiotic utilization [15].

There have been previous published descriptions of FilmArray use. However, few studies explored the clinical utility of the FilmArray ME panel in commonly encountered clinical cases of aseptic meningitis. We have previously reported the role of molecular diagnostics in the area of infectious disease diagnostics with actionable case management [16, 17]. Therefore, in this study, we analysed the detection of the different targets of the FilmArray ME panel and observed clinical outcomes where its utility has superseded Gram staining in a routine laboratory. Further, our study describes large scale use, including discordant results.

## Methods

### Setting and inclusion criteria

This retrospective study was conducted in University Hospital Limerick (UHL), a 455-bed tertiary referral centre in the Mid-West of Ireland, serving a population of 473,000, part of the University Limerick Hospital group (ULHG). All routine CSF testing was performed in the clinical microbiology laboratory located in UHL. All patients who had cerebrospinal fluid (CSF) obtained via a lumbar puncture for testing with the FilmArray ME panel over a 3-year period, from October 2017 to October 2020, were included in the study. Our centre does not provide specialised quaternary neurosurgical services.

### Ethical approval

This study was approved by the Research Ethics Committee of University Limerick Hospital Group, Limerick, Ireland. All data accessed were anonymised and individual patient consent deemed not required.

### CSF testing

All CSF samples arriving in the microbiology laboratory are processed immediately on arrival. Macroscopic appearance of the CSF is reported. UHL laboratory protocols for CSF testing are similar to those reported previously in our study of the FilmArray ME panel [18]. Leucocytes and erythrocytes were quantified by manual microscopy using a KOVA Glasstic Slide counting chamber in which 1μl of CSF was assessed using manual light microscopy. 10μl of uncentrifuged samples were inoculated on two 5% sheep blood agars (Oxoid) incubated in 5% CO_2_ at 35–37° and also in an anaerobic atmosphere. In addition, a chocolate agar (Oxoid) was incubated in 5% CO_2_ at 35–37° for 48 hours. Subsequent to our previous study outcomes [18], Gram staining of CSF is no longer included in routine testing. All CSF samples with >5 leucocytes/μL underwent analysis using the FilmArray ME panel. For CSF with ≤5 leucocytes/μL the FilmArray ME panel was performed on request following discussion with the Medical Microbiologist, where appropriate clinical indication was found to be present, for example where clinical features were consistent with meningo-encaphalitis despite a normal leucocyte count or suspicion of CNS infection in an immunocompromised individual. Processing for the FilmArray ME panel was performed as per manufacturer’s instructions. In summary, approx. 200 μL CSF is lysed using provided buffers prior to qualitative PCR using proprietary primers on the *in vitro* FilmArray® Multiplex PCR analyser. The entire PCR process occurs within one pouch and takes approx. 1 hour from start to completion.

Any corroboratory testing required involved sending samples to the Irish National Virus Reference Laboratory (NVRL) for confirmation of viral pathogens and to the Irish Meningococcal and Sepsis Reference Laboratory (IMSRL) for bacterial pathogens. Both utilise PCR-based testing. Confirmatory testing during the study period was on a request basis by the Medical Microbiologist following discussion with the attending physician.

The primary objective of our study was to observe the performance of the FilmArray ME panel for bacterial and viral targets. The sensitivity and specificity values for viral targets were calculated for samples that underwent duplicate testing with reference laboratory PCR methods. We were not able to compare the bacterial results with reference laboratory performance as insufficient samples were referred for molecular testing. For all discordant results, radiological imaging, electronic and paper-based records were reviewed by the authors to determine the significance of the result.

### Patient outcome data

The secondary outcome assessed in this study is the clinical outcomes for patients diagnosed as having bacterial meningitis, viral meningoencephalitis, or aseptic meningitis. We compared the three groups to assess the clinical value of the assay in cases of aseptic meningitis by comparing patient outcomes. For standardization, the definition of aseptic meningitis used is the presence of clinical evidence of meningitis accompanied by a CSF pleocytosis, defined as a white cell count (WCC) of >5 per μl (when corrected for number of red cells in the CSF of 1:500), and a negative FilmArray ME panel, which is modified from a previous international working group which uses a negative Gram stain as one of the criteria [19]. Comparative outcomes for 30 and 90-day all-cause mortality and 90-day all-cause readmission to hospital were examined for confirmed bacterial meningitis, viral meningoencephalitis or aseptic meningitis based on the FilmArray ME panel. Admission to a critical care area is defined as admission to the intensive care unit (ICU), high dependency unit (HDU), neonatal ICU or the paediatric HDU.

Data for CSF samples tested using the FilmArray ME panel were collected retrospectively via the laboratory information management system (DXC/iLAB). Data specific to patient episodes and admissions were identified and retrieved using the hospital’s electronic Inpatient Manager System (iPMS). Clinical data pertinent to final diagnoses were obtained via laboratory electronic notes, those compiled by the Medical Microbiology team, Emergency Department notes, radiological reports, and paper-based medical records. All patient data were anonymised in compliance to the General Data Protection Regulation (GDPR).

### Statistics

Analyses were performed using SPSS v26.0 (IBM) and p-values were calculated to ascertain statistical significance. A p-value of ≤0.05 was considered statistically significant. For categorical data, Kruskal-Wallis analysis was performed. For continuous data, comparison was conducted using one-way ANOVA.

## Results

**Fig 1:**
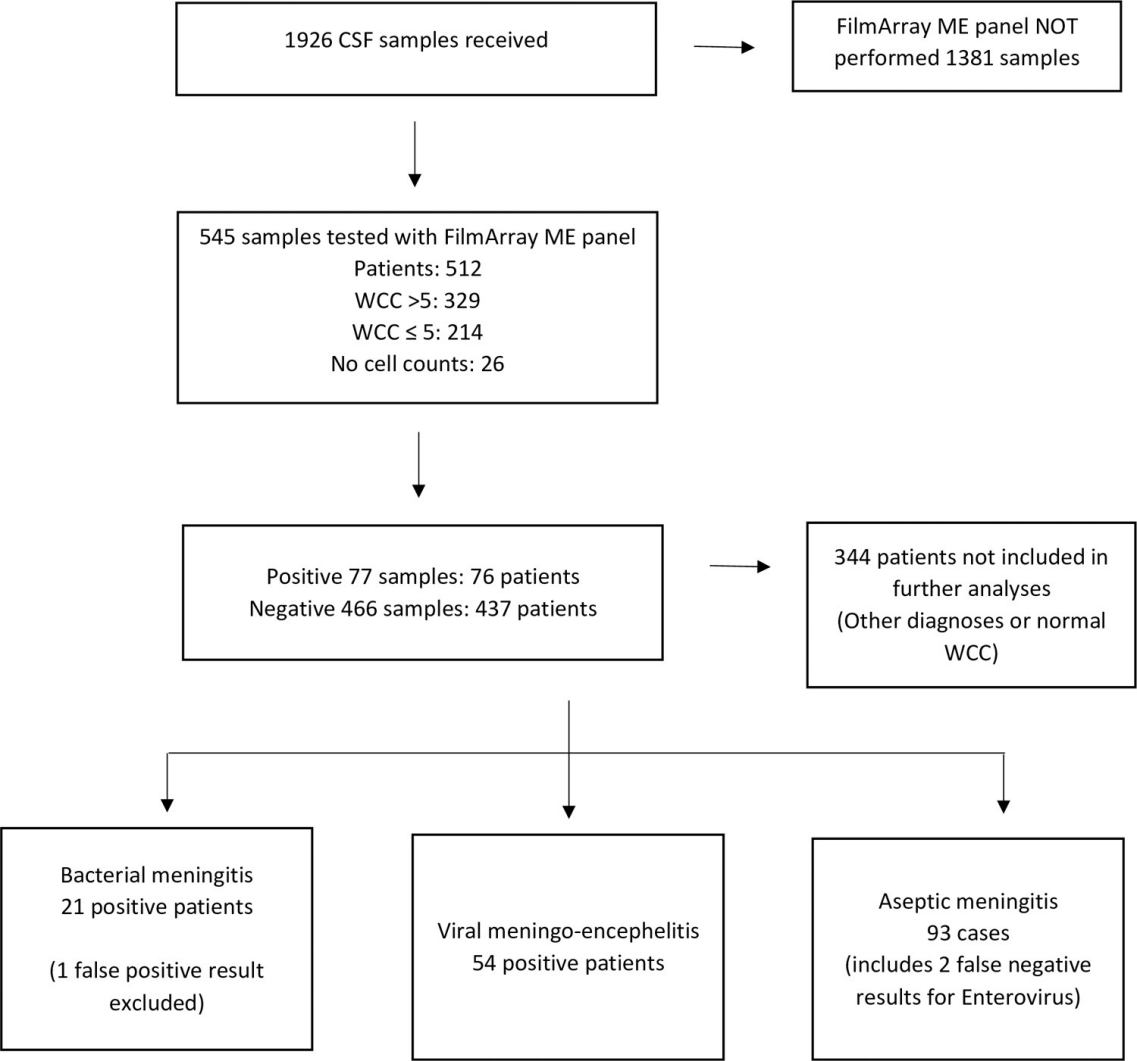
CSF samples and patients during study period.

From October 2017 to October 2020, 1926 CSF samples were received in the Clinical Microbiology laboratory (Fig 1). Of those, 543 CSF samples from 512 individual patients were tested using the FilmArray ME panel. An abnormal WCC was noted for 329 samples (60.6%); no WCC was available for 26 blood-stained samples. A positive result was detected in 78 (14.4%) of all samples. Bacterial pathogens were detected in 22 samples and viral pathogens in 56 samples. In 466 samples, no target pathogen was detected. There was no positive result for *Cryptococcus neoformans* either via FilmArray ME panel, cryptococcal antigen testing or culture methods. Six CSF samples were positive for culture (2 beta-haemolytic group B streptococci, 3 *Streptococcus pneumoniae* and 1 *Escherichia coli*). Of 21 confirmed bacterial meningitis cases in our study, all patients had received at least one dose of antibiotic at the time of lumbar puncture. Positive results from the FilmArray ME panel are provided in Table 1. 103 CSF samples were referred to the NVRL, including confirmatory testing of viral targets in eight positive specimens, and a further 95 FilmArray-negative specimens were sent based on clinical suspicion of viral meningoencephalitis despite a negative FilmArray ME panel result, with particular emphasis on corroboratory testing with respect to suspicion of HSV-1 or 2 meningoencephalitis. Of importance, no bacterial pathogens were found on culture on the remaining 1383 samples that did not undergo molecular diagnostics.

**Table 1:** Positive results for FilmArray ME panel in study.

Target	FA Positive	Culture Positive	Samples referred to the NVRL	PCR Confirmed NVRL	Discordant FA positive, NVRL neg	Discordant FA negative, NVRL positive	Concordant positive FA/NVRL	Concordant negative FA/NVRL
*Streptococcus pneumoniae*	10	3	N/A	N/A	N/A	N/A	N/A	N/A
*Neisseria meningitidis*	4	0	N/A	N/A	N/A	N/A	N/A	N/A
*Listeria monocytogenes*	2	0	N/A	N/A	N/A	N/A	N/A	N/A
Group B beta-haemolytic streptococci	4	2	N/A	N/A	N/A	N/A	N/A	N/A
*Haemophilus influenzae*	1	0	N/A	N/A	N/A	N/A	N/A	N/A
*Escherichia coli*	0	1	N/A	N/A	N/A	N/A	N/A	N/A
Enterovirus ^a^	22	N/A	103	2	0	2	0	99
*Varicella-zoster* virus ^a^	16	N/A	103	1	0	0	1	102
Human herpes virus 6^a^^,^^c^	10	N/A	19	2	0	0	2	17
Herpes simplex virus (HSV) 1^a^	1	N/A	103	2	1	2	0	100
Herpes simplex virus (HSV) 2 ^a^	3	N/A	103	0	0	0	0	103
Human parechovirus^a^^,^^c^	2	N/A	19	2	0	0	2	17
Enterovirus & human herpesvirus 6^b^	2	N/A	N/A	N/A	N/A	N/A	N/A	N/A
Cytomegalovirus ^a^^c^	0	N/A	6	2	0	2	0	4

FA, FilmArray ME panel; NVRL, National Virus Reference Laboratory

^**a**^ Only a limited number of samples were sent for corroboratory testing in the NVRL based on clinical need as determined by the Medical Microbiologist.

^b^ Two samples tested with 2 positive targets on the FimArray ME panel. Both samples were not referred for any corroboratory testing.

^c^ Enterovirus, HSV-1, HSV-2 and VZV is routinely performed for samples referred to the NVRL. Human parechovirus and HHV-6 is only tested in samples for patients under the age of 1 or upon special request. CMV is only available upon special request.

In the study, there were 2 confirmed false positive and 8 false negative results when the FilmArray ME panel were compared to reference laboratory results. Two false positive results were confirmed in our study, one for *Streptococcus pneumonia* and one HSV-2. Our study had eight false negative results: 2 enterovirus; 1 *Varicella-zoster* virus; 2 cytomegalovirus (CMV); 2 HSV-1; and 1 *E*. *coli*. Table 2 further describes these cases, including the clinical case and reference laboratory results. In our study, on further investigation of the false negative CMV results our reference laboratory limit of detection (LOD) was superior to the FilmArray ME panel, which explained the discordant result. The LOD of the FilmArray ME panel for CMV was 4300 copies/ml, far in excess of 57 copies/ml for the Artus CMV PCR assay (Qiagen, Hilden, Germany) performed in the NVRL [20].

**Table 2:** False positive and negative results: Clinical history, laboratory results and interpretation.

	Clinical history	WCC/μl	RCC/μl	PMN/ Mononuclear cell ratio (%)	CSF culture	FilmArray ME panel result	Reference laboratory and other relevant results	Interpretation
1	66-year-old female, presented with headaches and generalised tonic-clonic seizures with severe hyponatremia.	69	<10	1/99	No growth	*Streptococcus pneumoniae*	*Streptococcus pneumoniae* ND on lab-developed PCR (IMSRL)	False positive, non-reproducible result on culture, reference laboratory PCR and repeat FilmArray on same sample.
2	28-year-old female admitted with flare of relapsing-remitting multiple sclerosis. No features of meningoencephalitis.	32	1728	Not performed	No growth	HSV-2	Not detected for HSV-2 (Altona Realstar CNS screen) NVRL	False positive, symptoms attributable to multiple sclerosis.
3	42-year-old male, presented with headache and signs of meningism, with no significant medical history.	12	132	0/100	No growth	ND for all targets	CMV detected <500 copies/ml on Artus CMV PCR kit (NVRL), CMV IgM positive	False negative due to levels below the limit of detection for CMV but significance uncertain as patient recovered fully without CMV specific treatment. Treated as an aseptic meningitis.
4	3-day old neonate admitted to NICU with features consistent with congenital CMV infection.	<5	451	Not applicable	No growth	ND for all targets	CMV detected <500 copies/ml on Artus CMV in NVRL, CMV DNA detected in urine	False negative due to levels below the limit of detection for CMV.
5	59-year-old male presented with Miller-Fisher syndrome. Patient had a diagnosis of Bell’s palsy 2 weeks prior to acute admission.	<5	<10	Not applicable	No growth	ND for all targets	VZV weakly detected on FTD viral meningitis panel (FastTrack Diagnostics, Luxembourg) but not confirmed on LDT in NVRL.	Possible false negative or non-specific reaction with reference laboratory assay. Postulated possible Ramsay-Hunt syndrome 2 weeks prior to LP.
6	11-day old neonate presented with herpetic lesion on scalp. Treated as disseminated HSV-1 infection.	<5	3078	Not applicable	No growth	ND for all targets	HSV DNA detected on FTD viral meningitis panel in NVRL.	HSV-1 in CSF was below limit of detection of FilmArray ME panel.
	Repeat lumbar puncture performed at day 21 of IV acyclovir treatment.	15	39	10/90			HSV-1 detected on swabs of skin lesion, eyes, and oral mucosa.	Cycle threshold for PCR for 1st sample was 34.6 and 36.8 on repeat sample on FTD panel.
7	30-day old male infant admitted with generalised irritability.	40	<5	1/99	ESBL producing *E*. *coli*	ND for all targets	ESBL producing *E*. *coli* clean catch urine sample with similar antibiogram.	False negative due to non-K1 antigen *E*. *coli* isolate causing meningitis and urosepsis.
8	4-month-old female infant admitted with generalised irritability without clear signs or symptoms of meningitis.	<5	<10	Not applicable	No growth	ND for all targets	Enterovirus RNA detected in NVRL (Fast Track diagnostics). Enterovirus detected in plasma.	False negative. Enteroviral infection with self-limiting meningitis.
9	17-year-old male with previous brain tumour in remission, presented with 2-day history of headache and signs of meningism.	92	228	2/98	No growth	ND for all targets	Enterovirus RNA detected in NVRL (Fast track). Echovirus 30 on confirmation via LDT.	False negative. Echovirus 30 not within verified targets for FilmArray. Self-limiting enteroviral infection with no evidence of tumour recurrence on neuroimaging.

WCC, white cell count; RCC, red cell count; HSV, herpes simplex virus; ND, not detected; PCR, polymerase chain reaction; NICU, neonatal intensive care unit; CMV, cytomegalovirus; ESBL, extended-spectrum beta-lactamase; LDT, lab developed test.

For viral targets, the cumulative specificity was 98.9% (95% confidence interval: 93.1–99.9) and sensitivity was 53.8% (26.1–79.6) when compared to the reference laboratory methods. However, as only 13 positive FilmArray results for viral targets were referred for duplicate testing, sensitivity for viral targets should be interpreted with caution (Table 3). Due to the lack of a gold standard test for bacterial targets, we did not further assess the performance characteristics of bacterial targets.

**Table 3:** Specimens that underwent duplicate testing for viral targets.

		Reference Lab		
		Detected	Not Detected	
**FilmArray ME panel**	**Detected**	**7**	**1**	**8**
	**Not Detected**	**6**	**89**	**95**
		**13**	**90**	**103**

Four cases of mumps meningitis were diagnosed during the study period. In addition, two further cases of enterovirus meningitis were diagnosed via either stool or nasopharyngeal swab PCR result during the study period. The 6 cases were included in analyses for the aseptic meningitis group. In addition, 10 of 12 detected human herpes virus 6 (HHV-6) were not deemed clinically significant on review of related medical notes but were included in the analyses of results. Due to our agreed laboratory protocol for automatic use of the FilmArray ME panel in the setting of pleocytosis in the CSF, testing with the FilmArray ME panel was also performed on samples from patients with a variety of other final diagnoses including demyelinating disease, central nervous system malignancies (primary malignancies, lymphoma or metastatic disease), encephalopathies (hepatic and one urea cycle disorder encephalopathy), neurosyphilis, and amyloid angiopathy. No sub-analysis of this diverse range of diagnoses was performed.

### Patient outcomes

The median age of all patients included in the analysis was 34y (IQR 0 – 57y). The median age of viral meningoencephalitis patients was younger than bacterial and aseptic meningitis, although statistical significance was not reached (24y vs 38.5y and 34y, respectively, p = 0.133). A significantly higher proportion of patients with bacterial meningitis was admitted to critical care relative to viral or aseptic meningoencephalitis (66.7% vs 3.6% vs 7.6%, p = <0.001). There was no statistically significant difference across the three groups in 30-day and 90-day all-cause mortality. All patients with confirmed bacterial meningitis (n = 21) had received at least 1 dose of antibiotic prior to the lumbar puncture being performed.

The average length of stay was longer among the bacterial meningitis group versus the other two groups (20.5 vs 11.3 and 12.9 days, p = 0.009). A subgroup analysis of patients with a diagnosis of enteroviral meningitis with the FilmArray ME panel (n = 24) had the shortest average length of stay of 3 days. 90-day readmissions were higher among the aseptic meningitis group but was not statistically significant (15.1% vs 9.5% and 11.1% for bacterial and viral meningitis respectively, p = 0.722). Table 4 describes observed differences in clinical outcomes for the three groups.

**Table 4:** Patient characteristics and outcomes for 3 subgroups analysed.

	Bacterial	Viral	Aseptic meningitis	p value
Patients (n)	21	54	93	
Age (Median, IQR)	38.5 (0–60.5)	24 (0–46.5)	34 (20.5–46)	0.133
Gender n(%)				
• Male	12 (57.1)	27 (50)	46 (49.5)	
• Female	9 (42.9)	27 (50)	47 (50.5)	
CSF WCC				
• ≤5 n	0	18	0	
• >5 n	19	36	93	
• No count	2	2	0	
Mean	3230	319	315	
Median (IQR)	1711 (79–5427)	133 (15–460)	30 (10–85)	<0.001
ICU/NICU n(%)	13 (61.9)	2 (3.6)	2 (2.2)	<0.001
HDU n(%)	1 (4.8)	0	5 (5.4)	
30-day all-cause mortality n(%)	0	1 (1.8)	1 (1)	0.529
90-day all-cause mortality n(%)	1 (5)	1 (1.8)	1 (1)	
90-day readmissions n(%)	2 (9.5)	6 (11.1)	14 (15.1)	0.722
Average length of stay (days)	20.7	11.3	12.9	0.009
Median days (IQR)	16 (10.75–21)	7 (3–15.5)	8 (5–14)	

IQR, interquartile range; ICU, intensive care unit; NICU, neonatal intensive care unit; HDU, high dependency unit.

## Discussion

Utility of the FilmArray ME panel has been well established since it was first approved by the FDA in October 2016, and increasingly it is becoming a fundamental element of the clinical microbiology laboratory’s diagnostic portfolio. We observe its strength in diagnosing community associated bacterial meningitis in our hands. Our study found low mortality rates in patients with confirmed bacterial meningitis, due to early initiation of appropriate antibiotic therapy in this patient cohort. In comparison, Van de Beek et al. reported a mortality rate of 21% albeit that only patients with culture positive CSF were included [3]. In addition, only 9% of patients in their study had been pre-treated with antibiotics at the time of the lumbar puncture compared to 100% of patients with confirmed bacterial meningitis in our study receiving at least 1 dose of antibiotics prior to their lumbar puncture.

A further observed strength of the FilmArray ME panel is enabling of prompt decision making by clinicians, both in the context of positive and negative findings. This is evident as a diagnosis of self-limiting viral meningitis on the FilmArray ME panel, such as enteroviral meningitis, resulted in short admission stays. It is noteworthy the average length of admission for enterovirus meningitis was shortest amongst all the patient subgroups in this study. In our centre, prior to the introduction of the FilmArray ME panel, viral PCR testing had to be performed at the central Irish national reference laboratory with resultant delays in turn-around-time for results and impact on clinical care. This point is consistent with our previously reported positive use of the Abbott ID Influenza A + B as a near patient care testing device [17]. Furthermore, rapid time to reporting also completes the feedback loop to clinicians, which can lead to actionable case management and the importance of this point is well illustrated in Timbrooks et al. meta-analyses of the benefits of rapid molecular diagnostic on mortality in blood stream infection [21].

Our findings with regards to performance characteristics were more limited. Our calculated specificity for viral targets was 99% and similar to studies included in the meta-analysis of Tansarli and Chapin [14]. Due to the lack of any routine application of a recognised gold standard test for diagnosis of bacterial meningitis, we were unable to fully ascertain the performance characteristics for bacterial targets. Bacterial cultures could not be employed as a comparator against the FimArray ME panel based on recognition of cultures being inferior in this setting. However, we observe, with the exception of a single false positive and false negative described in Table 2, that all other bacterial targets detected were clinically significant. Hence, it remains reasonable to argue that the FilmArray ME panel has useful clinical application in bacterial meningitis diagnostics.

Separately in this study, we assessed the outcomes of a group of patients who were included as aseptic meningitis cases based on a modified criterion. Aseptic meningitis is an all-encompassing diagnosis that is not infrequently seen in clinical practice and has historically included both infectious (particularly viral) and non-infectious causes. We observe non-inferior clinical outcomes in the aseptic meningitis group compared to both the bacterial and viral meningitis cases. Thus, when taking into consideration the overall performance characteristic of the FilmArray ME panel, as derived from the meta-analyses by Tansarli and Chapin, with analyses of this subgroup in our study, a negative FilmArray ME panel outcome remains a useful result for clinicians who encounter cases of meningitis or meningism in clinical practice.

There is much debate regarding use of pleocytosis as a diagnostic criterion in use of the FilmArray ME panel. We chose pleocytosis of >5 WCC / mm3 as a threshold for automatic testing of CSF with the panel. This is consistent with what defines abnormal CSF WCC in the UK Standards for Microbiology Investigations of CSF [22]. Nonetheless, there remain limitations in using WCC as a screening criterion. In their studies of bacterial meningitis, Van de Beek et al. and Durant et al. described the lack of pleocytosis in 8.9% and 10%, respectively. However, it is noteworthy that pleocytosis was defined at a higher cut-off of ≥100 leukocytes/uL [3, 23]. Furthermore, a systemic review by Troendle and Pettigrew describes the occurrence of meningitis by bacterial, fungal and viral pathogens in the absence of CSF pleocytosis, albeit they concluded that this is a rare occurrence [24]. In our centre, the limitation of using CSF WCC as a testing criterion is offset by the availability of a 24 hour diagnostic liaison service led by a Medical Microbiologist for discussion of cases. These discussions account for the 34.7% of CSF with normal white cell count where the FilmArray ME panel was performed.

We report on three specific limitations of the FilmArray ME panel. Of particular importance, the discordant result on the FilmArray ME panel in a neonatal *E*. *coli* meningitis case with a negative FilmArray result and positive CSF culture. The FilmArray ME panel detects *E*. *coli* strains with K1 capsular polysaccharide only. Although *E*. *coli* isolates possessing the K1 capsular polysaccharide are dominant, an estimated 20% do not carry the specific target for the FilmArray ME panel [25]. This absence was confirmed to be the case in our neonatal isolate, resulting in a negative FilmArray result. Therefore, there is potential for false reassurance in a negative FilmArray result, especially if clinical suspicion of meningitis remains. Lee et al. reported a similar conclusion for their discordant result of a false negative result for *E*. *coli* in a Taiwanese study [26]. Hence, it is prudent to conclude that in cases of neonatal sepsis, treatment decisions need to be taken in tandem with CSF culture results. This finding is of clinical importance as the diagnosis of neonatal meningitis impacts treatment duration. Furthermore, in our study we observed the limit of detection for specific targets on the FilmArray ME panel to be inferior to other laboratory targeted real-time PCR assays, as discussed for our HSV-1 and CMV cases. In 2 of the 3 cases, the CSF testing was performed for neonate patients and the false negative result, due to the inferior limit of detection in the FilmArray ME panel, could potentially have provided false reassurance to clinicians and impact treatment decisions. We also briefly highlight the limitations of strain specific target detection given that another of our discordant result (Table 2, case 9) was caused by Echovirus 30 which is also not detected by the assay.

Our study also reemphasises the importance of clinical interpretation of results with regard to its clinical significance. Ten out of twelve positive results for HHV-6, of which 5 were confirmed by testing in the NVRL, were deemed not clinically significant based on clinical criteria and likely reflect chromosomal integration of the virus without evidence of meningoencephalitis [27]. One case with HHV-6 detected on the FilmArray ME panel was confirmed to be mumps meningitis which had mumps diagnostics not been performed, would have been erroneously deemed as a case as a HHV6 meningitis, which potentially could have delayed public health interventions. Boudet et al. and Redmard et al. reported similar conclusions in their studies with regard to HHV-6 [28, 29]. Clearly, the three issues highlight the importance of laboratories having robust communication processes to ensure limitations of any rapid molecular diagnostics introduced into routine clinical use do not impact negatively on clinical care. In our centre, a positive CSF for pleocytosis or a positive FilmArray ME panel target automatically triggers a discussion between the ordering clinician and the medical microbiologist to assist interpretation of results with a view to reducing the impact of any discordant results. This approach is supported by a recent study by Pandey et al. focused on diagnosis of HHV-6 meningitis in a paediatric centre [30]. In their study, twenty percent of HHV-6 positive results on the FilmArray ME panel was confirmed as HHV-6 meningitis, and review of cases by their Microbiologist on-call prior to reporting and phoning of result led to assisted interpretation of results and subsequent appropriate changes in antimicrobial prescribing.

Of interest, the four mumps cases in our study are consistent with notification of a national outbreak in Ireland starting in early 2018 and declining in April 2020, in part due to public health measures introduced secondary to the COVID19 pandemic [31]. Diagnosis of mumps was made via testing of CSF and buccal mucosa swabs performed in the NVRL, due to local clinical awareness of increased incidence of mumps in the community via national surveillance programmes. This supports the conclusion that the use of the FilmArray ME panel should be guided in conjunction with clinical and local epidemiological data, especially in regions where additional infective pathogens may be responsible for presentations of acute meningoencephalitis.

The limitations of our study relate to its retrospective design, and a causal relationship could not be confirmed between the FilmArray ME panel results and clinical outcomes for each individual group studied. We had no confirmed cases for *Cryptococcus neoformans* and cannot ascertain the performance characteristics for the FilmArray ME panel, which may limit generalisation of our findings in areas with elevated incidence of immunosuppression, for example in regions with high prevalence of HIV. Equally, our study findings may not be relevant to regions where other severe aetiologies of viral meningoencephalitis are endemic, for example, West Nile virus or Japanese encephalitis virus. This study was not designed to define morbidity following a diagnosis of bacterial meningitis, viral meningoencephalitis or aseptic meningitis, and may have missed any difference between the 3 groups. Further, due to the lack of electronic patient or prescribing records, the impact of the FilmArray ME panel on antimicrobial and antiviral prescribing for patients with suspicion of a CNS infection could not be confirmed. A cost analysis was not performed for the purpose of this study as this was completed for our previous study [18]. The cessation of Gram stain in our hospital was appropriate, considering only one bacterial target was missed. Nonetheless, this decision was based on local findings and may be context dependent with respect to the nature of the hospital, especially in neurosurgical centres. However, the outcome of this study remains widely applicable and may be a useful resource for clinical microbiologists and infectious disease specialists interested in establishing a new, or enhancing an existing, molecular diagnostic service.

In conclusion, the implementation of the FilmArray ME panel was a relative success in its diagnostic utility and can impact prudent clinical care. Our laboratory has been able to safely remove Gram stain as part of CSF testing, and ease of use of the FilmArray ME panel allows 24h, 7-day testing of abnormal CSF for CNS pathogens with a short turnaround time.
